# Current insights into monitoring of congenital adrenal hyperplasia

**DOI:** 10.3389/fendo.2026.1774145

**Published:** 2026-02-13

**Authors:** Quinty M. Leusink, Elke E. W. Verploegen, Bas P. H. Adriaansen, Xinyi Chin, Nike M. M. L. Stikkelbroeck, Paul N. Span, Fred C. G. J. Sweep, Margo Dona, Antonius E. van Herwaarden, Hedi L. Claahsen-van der Grinten

**Affiliations:** 1 Department of Pediatrics, Division of Pediatric Endocrinology, Amalia Children’s Hospital, Radboud University Medical Center, Nijmegen, Netherlands; 2 Department of Laboratory Medicine, Radboud University Medical Center, Nijmegen, Netherlands; 3 Department of Pediatrics, Endocrinology Service, KK Women’s and Children’s Hospital, Singapore, Singapore; 4 Department of Endocrinology, Radboud University Medical Center, Nijmegen, Netherlands; 5 Department of Radiation Oncology, Radboud University Medical Center, Nijmegen, Netherlands; 6 Department of Animal Sciences & Health, Institute of Biology, Leiden, Netherlands; 7 Department of Internal Medicine, Radboud University Medical Center, Nijmegen, Netherlands

**Keywords:** congenital adrenal hyperplasia (CAH), 21-hydroxylase deficiency (21OHD), monitoring, biomarkers, reference intervals

## Abstract

The management of 21-hydroxylase deficiency (21OHD), the most common form of congenital adrenal hyperplasia, remains challenging as both over- and undertreatment with hormone replacement therapy are associated with short and long-term complications. Monitoring of treatment efficacy typically combines clinical assessment with biochemical evaluation by measuring specific steroids. Currently, androstenedione and 17-hydroxyprogesterone are the most commonly measured biomarkers, and their concentrations are interpreted using available reference intervals. However, inter-center variation in the concentrations of these steroids has been observed, likely due to the heterogeneity in monitoring practices and analytical methods. Additional sources of variation include the selection of biological matrix, timing of sample collection relative to diurnal rhythm and medication administration, and interpretative challenges of biomarker levels. Age-dependent fluctuations in steroid concentrations, particularly within the pediatric population, underscore the necessity for age-specific reference intervals. This review evaluates current monitoring strategies and reported reference intervals, and explores emerging biomarkers, including 11-oxygenated androgens and indicators of glucocorticoid receptor sensitivity, along with non-invasive sampling approaches. Together, these developments may enhance the precision and ease of disease monitoring in patients with 21OHD. Overall, this review emphasizes the need for standardized monitoring practices and method- and age-specific reference ranges, aiming for assay harmonization and optimal disease control.

## Graphical Abstract

**Figure:**
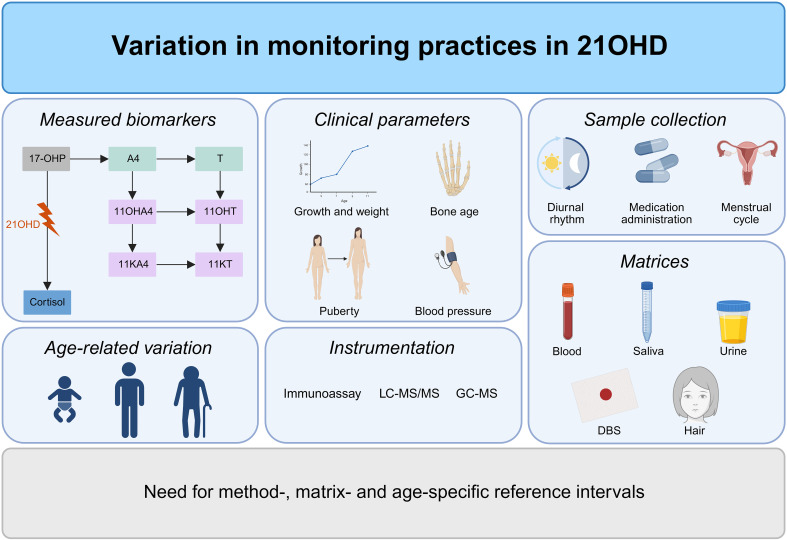

## 1 Introduction

Congenital adrenal hyperplasia (CAH) is a group of autosomal recessive disorders of the adrenal cortex caused by an enzyme deficiency involved in adrenal steroidogenesis, with a prevalence of 1:14,000 to 1:18,000 (1). In approximately 95% of the cases, 21-hydroxylase deficiency (21OHD) is the underlying cause of CAH (2). 21OHD is characterized by reduced or absent enzyme activity, leading to impaired cortisol synthesis. In the most severe phenotype, the salt-wasting (SW) form, minimal or no residual enzymatic activity (< 1%) results in near-complete deficiencies of both cortisol and aldosterone. The consequent loss of negative feedback triggers adrenocorticotropic hormone (ACTH) release of the anterior pituitary gland, driving chronic stimulation of the adrenal cortex, leading to adrenal hyperplasia and overproduction of adrenal androgens. As androgen concentrations are already strongly elevated *in utero*, female patients are born with a variable degree of virilization of the external genitalia (3). Without timely intervention, affected neonates can develop life-threatening SW crises (1). Apart from the SW form, the simple-virilizing (SV) form represents a less severe variant of 21OHD, characterized by residual enzymatic activity of 1-2% and largely preserved mineralocorticoid (MC) synthesis. Nevertheless, affected individuals still exhibit cortisol deficiency accompanied by accumulated adrenal androgen concentrations leading to virilization of external genitalia in girls (4).

In both SW and SV 21OHD, precursor steroids prior to the enzymatic block accumulate, e.g., 17-hydroxyprogesterone (17-OHP) and 21-deoxycortisol (21-dF). Neonatal screening, available in most developed countries, relies on measuring 17-OHP concentrations, and if available 21-dF levels as second-tier test (5). Following an abnormal screening result, the diagnosis is confirmed by measuring adrenal steroid precursors and androgens, genetic analysis or a stimulated adrenal function test, such as a cosyntropin test (6). The accumulated precursor steroids are partially shunted into the unaffected adrenal androgen pathway, resulting in androgen excess. This hyperandrogenism causes severe symptoms, including virilization of the female external genitals, precocious puberty, and menstrual irregularities. Consequently, patients require lifelong glucocorticoid (GC) therapy, aiming at both replacement of deficient cortisol and effective suppression of androgen excess by reducing ACTH-driven adrenal hyperplasia. During childhood and adolescence, treatment goals are supporting normal linear growth, achieving a final height within the target range, and ensuring normal pubertal and psychosexual development, in addition to cortisol substitution to prevent Addisonian crisis. Across the entire lifespan, key therapeutic objectives include maintaining a healthy body weight, ensuring optimal quality of life, preventing adrenal crises, optimizing fertility, and minimizing long-term complications (7). Patients with 21OHD are treated with supraphysiological GC doses to adequately suppress ACTH levels. Treatment must carefully balance efficacy and safety. Undertreatment fails to replenish cortisol deficits and allows androgen excess to persist, whereas overtreatment predisposes patients to adverse effects, such as osteoporosis, hypertension, obesity, insulin resistance, dyslipidemia and impaired glucose metabolism (8, 9). Regular monitoring is therefore crucial to optimize outcomes, enabling titration of GC and MC doses and maintaining androgen levels within the appropriate range (10).

The Endocrine Society published an international consensus statement on the management of CAH in 2018, providing recommendations for diagnosis, monitoring, and treatment (11). This guideline serves as an important foundation for harmonizing clinical practice across centers. As a complement to this guideline, practical guidance regarding sample matrices, measurement methodologies, and biomarker reference ranges would further enhance its utility. Bacila et al. (2021) and Lawrence et al. (2022) highlighted substantial inter-center variation in total hydrocortisone (HC) dosing and biomarker levels (12, 13). These studies demonstrated that treatment practices varied across institutions, with center-specific effects explaining approximately one-third of the observed variation in biomarker levels. Contributing factors include the use of different analytical methods, diverse sampling matrices, and inconsistent timing of sample collection. These findings indicate the importance of recognizing center-specific differences when interpreting and comparing biomarker results. Identifying and understanding the sources of variation could pave the way for harmonizing assays and monitoring practices, supporting the implementation of standardized thresholds. This review outlines current knowledge on biomarker interpretation and monitoring strategies in patients with 21OHD across childhood, adolescence, and adulthood. Ultimately, it emphasizes the need for age-, method-, and matrix-specific reference intervals to support optimal patient care.

## 2 Clinical biomarkers

During childhood and adolescence, growth is affected by different mechanisms. In patients with 21OHD, poor hormonal control with elevated adrenal androgen concentrations can advance linear growth while accelerating skeletal maturation, resulting in premature closure of epiphyseal plates and potentially compromising final height (14). In addition, over- and undertreatment with GC replacement and fludrocortisone (FC) may limit the ability to achieve normal growth. Therefore, growth is an important clinical indicator of long-term disease control in patients with 21OHD as this directly reflects the balance between androgen excess and GC exposure (4, 15). Consequently, normal linear growth is a primary objective of treatment monitoring in pediatric patients with 21OHD, which can be assessed by serial height measurements obtained at each clinical visit, plotted against age- and sex-specific growth charts (16). However, their interpretation must integrate chronological age, parental target height, and pubertal status to distinguish physiological variation from disease activity.

In addition to growth, skeletal maturation is routinely assessed, as insufficient suppression of adrenal androgen concentrations accelerates bone maturation. Bone age, typically determined via annual left-hand X-rays, is an important parameter for monitoring long-term disease control and predicting final height (11). In addition to their relevance in childhood, bone health remains an important consideration throughout life. Exposure to high GC replacement dosages is associated with decreased bone mineral density (BMD) and an increased risk of early-onset osteopenia or osteoporosis (17). A retrospective analysis demonstrated significantly lower whole-body BMD Z-scores in adult males with 21OHD compared with age-matched healthy controls (18). These findings highlight the importance of continuous bone health assessment to support optimal skeletal development and growth during childhood but also to mitigate long-term skeletal complications in adulthood.

Elevated androgen levels and supraphysiological GC exposure promote adiposity, increasing the risk of elevated BMI and obesity (19). The interplay between high BMI and metabolic dysfunction creates a self-reinforcing cycle, in which androgen synthesis promotes insulin resistance, while hyperinsulinemia further augments hyperandrogenism (20). Therefore, regular assessment of BMI is an essential component of monitoring patients with 21OHD.

Furthermore, high FC exposure increases the risk of hypertension, thereby advocating regular blood pressure monitoring. Neonates and young children are particularly susceptible to transient, FC-induced hypertension due to changing MC sensitivity (21). Nevertheless, blood pressure monitoring should be performed across the lifespan, as it provides a direct clinical measure to guide FC and GC therapy and prevent cardiovascular complications.

In pediatric patients with 21OHD, clinicians must closely monitor and support the induction of normal pubertal development. Elevated adrenal androgens can lead to precocious puberty, premature pubarche, breast development in girls or testicular enlargement in boys (1). Conversely, GC overtreatment may result in delayed puberty due to suppression of the hypothalamic-pituitary-gonadal (HPG) axis (22). As such, the promotion of normal pubertal timing is a central treatment objective in the management of 21OHD. Regular surveillance for early signs of both precocious and delayed puberty is essential to optimize pubertal development. Monitoring of gonadal function remains important throughout life to regulate normal menstrual cycles in females and preserve fertility in both sexes.

## 3 Biochemical biomarkers to monitor glucocorticoid treatment

Auxological parameters capture the cumulative impact of (sub)optimal treatment over extended periods. However, clinical evaluation should be supported by biochemical measurements to obtain the most robust assessment of disease control. Steroid measurements provide results of short-term fluctuations in adrenal hormone production. Hence, integrated monitoring that combines regular clinical assessment with biochemical measurements is essential to optimize clinical management, ensuring adequate androgen control while minimizing the adverse effects of overtreatment (11). To date, 17-OHP and androstenedione (A4) remain the most commonly used biomarkers for biochemical monitoring of disease control in patients with 21OHD. Impaired enzyme activity leads to accumulation of 17-OHP, which is generally considered the most direct indicator of upstream steroid excess. While 17-OHP is capable of transactivating the glucocorticoid receptor (GR), its intrinsic biological activity is limited due to its low binding potency compared with cortisol (23). Accumulated levels of 17-OHP are partly diverted into the androgen pathway, where A4 is directly derived from 17-OHP, making A4 an important marker of hyperandrogenism (24). A4 can undergo peripheral conversion to estrone and subsequently to estradiol. Estradiol, the most potent endogenous estrogen, plays a key role in skeletal maturation (25). Thus, elevated A4 levels contribute to increased estrogen synthesis, resulting in accelerated bone age advancement (10). In a retrospective study of 39 pediatric 21OHD patients, increased A4 levels correlated negatively with final height (26). Among post-pubertal patients without regular biochemical monitoring, higher 17-OHP levels were linked to lower height standard deviation scores (27). Both 17-OHP and A4 exhibit a diurnal pattern driven by ACTH secretion, which is consistent across sexes (24). Pre-pubertal testosterone (T) levels also correlate with 17-OHP, although this relationship is weaker than that observed for A4 (24). Consequently, A4 is considered a more specific and reliable indicator of adrenal androgen production in patients with 21OHD, demonstrating stronger associations with clinical outcomes (16, 28). Other androgen precursors, including dehydroepiandrosterone (DHEA) and dehydroepiandrosterone sulphate (DHEAS), are occasionally measured but provide limited value in 21OHD monitoring. In treated 21OHD patients, these steroids are typically suppressed to undetectable levels, while A4 levels may remain elevated. Therefore, DHEA(S) fail to discriminate between good and poor disease control (10). Accordingly, most centers currently employ A4 as a marker for hyperandrogenism, alongside 17-OHP measurements and clinical evaluation to assess treatment efficacy and long-term outcomes, as reflected in international guideline recommendations (11).

Reference ranges for 17-OHP and A4 have been assessed to facilitate accurate interpretation of biochemical markers and guide treatment monitoring in 21OHD patients. In general, A4 levels above the normal upper limit reflect poor control and should therefore be maintained within age-adjusted limits. In a cohort study of 122 21OHD patients, serum A4 levels of < 5 nmol/L were associated with good control (24). By contrast, normalization of 17-OHP levels is not advised, as this indicates GC overtreatment (11). Defining a precise target range for 17-OHP concentrations is essential to minimize the risk of overtreatment (15, 29). Earlier studies indicated that adequate control was achieved when A4 levels were within the normal range with serum 17-OHP at supraphysiological concentrations of < 36-38 nmol/L (24, 30, 31). Consistent with these findings, Troger et al. (2022) observed that high 17-OHP concentrations (> 30 nmol/L) were associated with a lower estimated final height (32). These findings emphasize the importance of lowering 17-OHP levels, while avoiding the adverse effects of GC overtreatment (33).

In general, 17-OHP and A4 exhibit a strong linear correlation (12). However, discrepancies between these two biomarkers are not uncommon in clinical practice, in which one marker is excessively elevated exceeding the acceptable range while the other remains within the reference range, thereby complicating the interpretation of biochemical disease control. In a U.S. cohort, Jha et al. (2021) reported discrepant 17-OHP and A4 levels in 17% of laboratory assessments. In this cohort, 86% showed A4 concentrations within reference range with significantly elevated early-morning 17-OHP levels, of which more than half demonstrated good clinical control according to clinical evaluation (34). Variations in biomarker levels and cutoff thresholds may be attributed to multiple factors, i.e., medication type and dosage, diurnal rhythm, age-dependent physiological changes, differences in measurement methods and analytical techniques, and diverse sample matrices. Furthermore, both 17-OHP and A4 present with interpretative challenges. 17-OHP fluctuates during the menstrual cycle of females, while A4 reflects both adrenal and gonadal steroid production, complicating its interpretation from puberty onwards, and exhibits an age-related decline in both sexes (35, 36). These sources of variation underline the challenges of accurately interpreting these biochemical markers in patients with 21OHD.

## 4 Age-dependent variation in steroid biomarkers

Accurate interpretation of steroid measurements in patients with 21OHD requires a thorough understanding of age-related hormonal variation. Adrenal and gonadal steroidogenesis undergo dynamic changes across developmental stages, necessitating the use of age-specific reference intervals to guide diagnosis, monitoring, and treatment.

During intrauterine development, the fetal adrenal gland is characterized by a definitive zone (DZ) and a fetal zone (FZ), with a transition zone (TZ) appearing during mid-gestation. Fetal adrenals mainly produce DHEA(S), serving as a precursor for placental estrogen synthesis. Around 8-9 weeks post-conception, cortisol production peaks in healthy individuals, coinciding with the critical window of sex differentiation (37). This rise in cortisol establishes a negative feedback mechanism that suppresses ACTH secretion, thereby restraining androgen synthesis and supporting typical female development. The backdoor pathway, an alternative route of androgen synthesis, contributes to the total androgen pool in patients with 21OHD. Activation of this pathway may be initiated prenatally, where excess 17-OHP is diverted toward potent androgens such as 11-ketodihydrotestosterone (11KDHT) (38). Following birth, the FZ involutes rapidly, reducing the adrenal size by half causing a marked decline in DHEA levels. Concurrently, 17-OHP levels are physiologically elevated in the first 24-48 hours of life compared with later stages (39). This increase is specifically pronounced in pre-term infants due to immaturity of adrenal steroid metabolism and in term neonates under stress-related conditions. Hence, early newborn screening (NBS) for 21OHD often results in a high false-positive rate when solely focusing on 17-OHP in combination with the use of low cutoff values to maximize sensitivity (39, 40).

Diurnal rhythm of cortisol varies significantly between individuals in the first six months (41–43). Adrenal androgen secretion remains quiescent in healthy children until adrenarche, which typically occurs between the age of 6 to 8 years. This phase is marked by the activation of the zona reticularis (ZR) and upregulation of CYB5A and SULT2A1, driving DHEA(S) production. Pubertal maturation is associated with increasing gonadal contribution to the total androgen pool in both sexes. In males, testicular T rises due to the activation of the HPG axis around 12 years of age, becoming the primary androgen source. In girls, ovarian androgenesis increases more gradually but still contributes to the circulating androgens (44). Salivary 17-OHP and A4 levels also rise during puberty, accompanied by evolving diurnal patterns (45). This complicates disease monitoring for pubertal and post-pubertal patients with 21OHD. Treatment goals must carefully balance suppression of adrenal androgen accumulation with preservation of normal growth, pubertal progression and fertility, while also accounting for the changes in cortisol pharmacokinetics (12, 46). Ageing is associated with a gradual decline in classic androgen levels, reflecting reduced testicular production in males and regression of the ZR in both sexes. This decline is more pronounced in postmenopausal women, where serum levels of DHEA(S) and A4 are reduced (36). Thus, production of steroids is highly age-dependent, influenced by many processes in life.

## 5 Biochemical monitoring of mineralocorticoid treatment

Aldosterone deficiency is present in roughly three-quarters of classic 21OHD patients, and even patients with residual aldosterone secretion benefit from FC by permitting lower GC doses to achieve adrenal suppression (47). MC replacement is therefore an essential component in the management of classic 21OHD. Careful monitoring of MC therapy is crucial as undertreatment leads to hyponatremia, hyperkalemia, salt cravings, dehydration, volume depletion with an increased risk of SW crisis, and failure to thrive, while overtreatment can result in hypernatremia, hypokalemia, hypertension, edema, and long-term cardiovascular complications (48). These challenges are particularly pronounced in neonates, in whom renal tubular immaturity leads to partial aldosterone resistance, necessitating higher FC dosing and supplemental sodium to prevent hyponatremia (11). As this physiological aldosterone resistance wanes with maturation within the first months of life, FC requirements generally decline (21, 49). Consequently, monitoring of MC replacement is essential during this period, as physiological aldosterone resistance and rapid developmental changes demand frequent dose adjustments.

MC treatment is commonly guided by blood pressure measurements, hydration status, and serum electrolytes, but these are late indicators of MC adequacy (50). Aldosterone production is regulated by the renin-angiotensin-aldosterone system and therewith driven by renin levels. When sodium concentrations decrease and renal perfusion reduces, renin production is increased. Measuring renin provides a more direct physiological biomarker of MC treatment, which resulted in the introduction of plasma renin activity (PRA) and later plasma renin concentration (PRC) immunoassays (51). In practice, elevated renin indicate the need to increase MC replacement, whereas suppressed renin suggests overtreatment (52, 53). A multicenter study of a U.S. cohort of 180 children with 21OHD reported an association between suppressed renin and hypertension, strengthening the practical target of maintaining renin in the high-normal range during MC titration (54). Specifically, PRA targeted in the upper reference range has shown to be a useful marker for optimal FC dosing (55). PRA and PRC measure distinct biological properties, i.e., enzymatic activity versus active renin mass. Hence, PRA and PRC are not numerically interchangeable, and their interpretation is affected by pre-analytical factors, e.g., dietary sodium intake, body position, time of day, and recent physical activity. Notably, assay choice may influence clinical decision-making. In a center transitioning from PRA to PRC, clinicians were more likely to increase FC dose when interpreting PRC, implying that assay-dependent interpretation may introduce inter- and intra-center variation (56). Standardized sampling and assay-specific interpretation are therefore essential to maintain consistency in longitudinal care and preserve clinical expertise. Although precise MC management is critical in infants, age- and assay-specific pediatric renin reference intervals remain lacking. For optimal MC monitoring, renin measurements should be paired with routine blood pressure monitoring and serum electrolyte measurements to guide clinical decisions (11, 57).

## 6 Biochemical monitoring of gonadal function

Pubertal development and gonadal function in patients with 21OHD requires careful monitoring of gonadal markers. During puberty, the gonads start to contribute to the total steroid pool due to the activation of the HPG axis. Disturbances in the establishment or regulation of the HPG axis can affect pubertal timing, sexual maturation, and fertility.

In females, ovarian steroid hormones, particularly estrogen and progesterone, exert feedback control on the HPG axis to maintain regular menstrual cycles. From puberty onwards, increased production of progesterone and adrenal androgens in poorly controlled patients with 21OHD can disturb this feedback mechanism and alter gonadotropin-releasing hormone (GnRH) pulsatility and subsequently reduce luteinizing hormone (LH) secretion, resulting in anovulation and menstrual irregularities. Women with 21OHD with adequate hormonal control have LH pulse frequencies comparable to healthy controls, highlighting the importance of hormonal regulation for normal pubertal development and fertility (58).

In 21OHD females with no desire for pregnancy, combined oral contraceptive pills could have beneficial effects on symptoms of hyperandrogenism. Oral contraceptive pills modulate steroid production and reduce androgen bioavailability, thereby contributing to the reduction of acne and hirsutism. Reduction of androgen concentrations occurs primarily due to increased hepatic synthesis of SHBG, resulting in decreased concentrations of free circulating androgens. Furthermore, the progesterone component of combined oral contraceptive pills suppress LH secretion, thereby reducing ovarian androgen production (59). However, in women with 21OHD that seek motherhood, decreasing adrenal progesterone and 17-OHP oversecretion is essential for restoration of menstrual cycles (60). Elevated levels of progesterone may inhibit follicular growth and endometrial proliferation, negatively influencing the likelihood of conception. Therefore, in addition to normalization of androgen levels, reduction of progesterone concentrations below 2 nmol/L has been demonstrated to enable ovulation and fertilization (11). This can be achieved by increasing GC and FC dosages. Casteras et al. demonstrated that administration of prednisolone in a thrice-daily regimen was most effective in reducing progesterone levels and enabling conception (61).

Similar mechanisms are observed in males, in which adrenal androgens and progestogens suppress the HPG axis, influencing gonadal function (10, 62). In poorly-controlled 21OHD males, elevated A4 levels suppress gonadotropins, resulting in a high A4/T ratio (>2), whereas in well-controlled patients, T production originates mainly from the testes with minimal adrenal contribution, resulting in a low A4/T ratio (<0.5) (63). Poor hormonal control is also associated with the development of testicular adrenal rest tumors (TARTs) (64). Although TARTs are often benign and lesions below 2 cm are generally not detected by palpation, these can damage testicular Leydig and Sertoli cells and cause primary hypogonadism and infertility. Measuring LH and FSH levels help in distinguishing between primary hypogonadism, caused by TART, and secondary hypogonadism, due to poor hormonal control, thereby providing complementary information on HPG axis function. The biochemical profile of an untreated adult 21OHD patient typically reveals suppressed or normal gonadotropins with low-normal T levels, accompanied by reduced inhibin B levels (62). Therefore, solely measuring gonadotropins is less valuable to evaluate gonadal function in 21OHD patients, as normal levels of gonadotropins and T may be detected despite gonadal dysfunction. Hence, inhibin B could serve as a complementary biomarker for Sertoli cell function, due to its correlation with spermatogenesis (65).

Overall, integrated monitoring that combines adrenal-derived steroid biomarkers, gonadal hormones, gonadotropins, and inhibin B is essential to guide normal pubertal development and fertility. This approach allows clinicians to evaluate effects of androgen excess on pubertal progression and prevent long-term gonadal complications.

## 7 Variation in measurement methods

Laboratories use a variety of instruments, measurement methods, and protocols for biochemical assessments. These differences hamper direct application of reference values across analytical methods.

Immunoassays have been the most commonly used method due to their simplicity, rapidity, widespread availability, and suitability for high-throughput screening. As a result, immunoassays are often used to measure 17-OHP in NBS samples (11). However, these assays rely on antibody-antigen interactions, which could lead to cross-reactivity with structurally similar steroids, often resulting in overestimation of hormone concentrations (66, 67). Consequently, immunoassays have limited specificity for 21OHD, where precursor steroids attain significantly elevated levels in comparison to healthy individuals. In cortisol immunoassays, often used for cosyntropin stimulation testing, cross-reacting precursors may cause such extensive overestimation that true cortisol deficiency remains undetected. In addition, measurement of low-abundance steroids such as T in females and prepubertal males is often compromised. Immunoassays failed to detect subtle adrenarchal rises in A4 and measured inconsistent T levels (67, 68). This complicates the use of immunoassays for optimal monitoring of patients with 21OHD.

Liquid chromatography-tandem mass spectrometry (LC-MS/MS) offers several advantages over immunoassays. This technique provides high analytical specificity by separating analytes based on polarity and their fragments on mass-to-charge ratio, thereby minimizing interference from other structurally-related steroids. This approach enables multiplexing, allowing simultaneous quantification of a comprehensive steroid profile. Its high sensitivity makes this technique well-suited for quantification of low-concentration samples, such as in neonates. Speiser et al. described that approximately 40% of NBS samples reported as positive for 21OHD by immunoassay were, in fact, false-positives, displaying normal 17-OHP levels when analyzed by LC-MS/MS, underlining the superior analytical specificity of LC-MS/MS (11). Despite its clear advantages, considerable variation in results and reported reference ranges has been observed among laboratories employing LC-MS/MS, suggesting that factors such as sample preparation, methodology and calibration standards may influence steroid measurements (36, 44, 69). Moreover, LC-MS/MS requires specialized and expensive equipment as well as technical expertise to ensure accurate and reproducible results.

## 8 Evaluation of reference intervals

Numerous research groups have established reference ranges for both classic androgens and 11-oxygenated androgens (11OA) measured by LC-MS/MS, predominantly in serum. However, reported values often show substantial differences across studies, reflecting differences in instrumentation, methodology, sample processing, matrix choice, age stratification, and population characteristics, as outlined in this review (**Table 1**). When analytical methods are comparable and population characteristics are similar, reference intervals can often be transferred from external centers. The comparability of measurement methods across the measuring range can be evaluated by a method comparison study. Method comparison is applicable when the laboratory employs an analytical method that is identical or highly comparable to that of another center. In this approach, a set of patient samples is analyzed using both methods, allowing for a direct comparison of results (74). Subsequently, a verification study involving samples from healthy individuals could confirm whether the reference values are appropriate for center’s patient population. According to international guidelines, measuring 20 healthy reference subjects provides sufficient evidence to assess the applicability of externally derived reference intervals. If at least 18 of the 20 results fall within the proposed range, the reference interval is considered verified, which corresponds to a probability of false rejection of approximately 5-7% (75). Through these verification procedures, laboratories can implement externally derived reference intervals in their own analytical and clinical setting.

**Table 1:** Reported human serum reference intervals of classic androgens and 11-oxygenated androgens measured by LC-MS/MS.

	Population	DHEA	A4	T	DHT	11OHA4	11KA4	11OHT	11KT
Adult population									
Schiffer et al. (2023) (36)	48 men < 50 years	8.1 [2.5-12.0]	1.5 [<0.70-5.0]	13.0 [8.3-19.0]	1.4 [0.60-2.3]	7.6 [2.9-14.0]	3.5 [1.42-6.2]	0.47 [<0.33-1.1]	0.86 [<0.33-2.2]
	76 men ≥ 50 years	5.0 [1.5-12.0]	1.7 [<0.70-3.2]	12.0 [6.5-22.0]	1.1 [<0.34-2.2]	8.5 [4.6-16.0]	2.1 [0.60-5.2]	0.72 [<0.33-1.5]	0.81 [0.36-1.8]
	84 women < 50 years	9.6 [4.2-20.0]	2.3 [0.71-5.3]	0.61 [<0.35-1.2]	<0.34 [<0.34-0.80]	7.5 [3.4-14.0]	3.2 [1.6-5.4]	0.39 [<0.33-0.78]	0.70 [<0.33-1.6]
Davio et al. (2020) (69)	81 women ≥ 50 years	5.0 [1.3-16.0]	1.1 [<0.70-2.9]	0.45 [<0.35-1.2]	<0.34 [<0.34-0.36]	8.4 [3.9-16.0]	1.8 [0.64-4.2]	0.63 [<0.33-1.2]	0.94 [0.37-1.7]
	69 men age 18-39	n.m.	1.7 [1.36-2.09]	16.4 [11.8-20.8]	n.m.	4.1 [3.1-5.6]	0.57 [0.43-0.70]	0.43 [0.36-0.56]	1.0 [0.66-1.4]
	99 men age 40-59	n.m.	1.7 [1.33-2.16]	15.0 [11.7-20.0]	n.m.	4.9 [3.6-6.8]	0.60 [0.47-0.77]	0.53 [0.40-0.76]	1.0 [0.63-1.3]
	108 men age 60-79	n.m.	1.4 [1.08-1.81]	16.2 [11.6-20.6]	n.m.	4.7 [3.6-5.9]	0.47 [0.40-0.67]	0.53 [0.36-0.70]	0.79 [0.60-1.1]
	43 men ≥ 80 years	n.m.	1.0 [0.91-1.33]	10.6 [6.2-15.8]	n.m.	4.7 [3.0-6.4]	0.43 [0.30-0.67]	0.50 [0.26-0.70]	0.63 [0.36-1.1]
	72 women age 18-39	n.m.	3.1 [2.3-4.05]	1.18 [0.80-1.42]	n.m.	3.9 [2.8-5.0]	0.50 [0.40-0.70]	0.36 [0.23-0.53]	0.70 [0.53-1.1]
	78 women age 40-59	n.m.	1.8 [0.63-1.17]	0.80 [0.66-1.08]	n.m.	4.0 [2.8-5.7]	0.50 [0.37-0.67]	0.40 [0.30-0.60]	0.86 [0.60-1.0]
	81 women age 60-79	n.m.	1.2 [0.84-1.54]	0.73 [0.52-1.18]	n.m.	4.1 [3.4-6.1]	0.50 [0.37-0.67]	0.50 [0.36-0.80]	0.86 [0.63-1.2]
	40 women ≥ 80 years	n.m.	0.9 [0.63-1.17]	0.73 [0.45-1.04]	n.m.	4.2 [2.9-5.7]	0.40 [0.30-0.57]	0.40 [0.26-0.60]	0.70 [0.46-1.0]
Nanba et al. (2019) (70)	100 women age 20-40 years	8.1 [5.4-14.0]	3.4 [2.64-4.86]	1.0 [0.76-1.39]	n.m.	5.7 [3.9-8.64}	1.2 [0.87-1.83]	0.46 [0.3-0.76]	0.86 [0.63-1.3]
	100 women age 60-89 years	2.7 [1.8-4.6]	1.2 [0.80-1.77]	0.7 [0.49-1.07]	n.m.	6.5 [4.67-9.57]	1.1 [0.77-1.53]	0.66 [0.43-0.93]	0.93 [0.73-1.22]
O**’**Reilly et al. (2017) (71)	49 women age 18-40 years	7.1 [4.2-11.8]	5.9 [3.3-9.2]	0.3 [0.2-0.5]	n.m.	6.8 [4.9-12.5]	2.7 [2.0-3.9]	0.2 [0.1-0.3]	1.5 [1.2-1.8]2.
Turcu et al. (2021) (72)	10 men age 19-29, circadian peak	n.m.	2.7 [2.7-2.8]	13.5 [13.0-14.0]	n.m.	8.6 [8.2-9.0]	2.1 [2.0-2.1]	0.6 [0.6-0.6]	1.4 [1.3-1.4]
	10 men, age 19-29, circadian nadir	n.m.	1.1 [1.0-1.2]	9.4 [8.7-10.1]	n.m.	0.7 [0.4-1.1]	0.4 [0.3-0.5]	0.0 [0.0-0.1]	0.3 [0.3-0.3]
	10 men age 61-75, circadian peak	n.m.	2.1 [2.0-2.1]	12.7 [12.2-13.3]	n.m.	7.3 [6.9-7.7]	1.5 [1.4-1.6]	0.5 [0.5-0.5]	1.0 [1.0-1.0]
	10 men age 61-75, circadian nadir	n.m.	1.0 [0.9-1.0]	9.6 [8.9-10.2]	n.m.	1.7 [1.4-2.1]	0.6 [0.5-0.7]	0.2 [0.2-0.2]	0.4 [0.4-0.4]
Caron et al. (2021) (73)	9 men age 21-62	8.2 ± 0.3	2.4 ± 0.1	18.7 ± 0.6	1.3 ± 0.0	4.4 ± 0.2	0.5 ± 0.0	0.6 ± 0.0	1.1 ± 0.3
	10 men age 60-72	4.9 ± 0.2	2.4 ± 0.1	19.6 ± 0.8	1.3 ± 0.1	6.8 ± 0.3	0.8 ± 0.0	0.5 ± 0.0	1.5 ± 0.1
	10 women, follicular phase age 20-40	11.2 ± 0.8	2.8 ± 0.1	0.6 ± 0.0	0.2 ± 0.0	5.3 ± 0.4	0.8 ± 0.1	0.3 ± 0.0	0.8 ± 0.1
	10 women, luteal phase age 20-40	9.4 ± 0.5	3.8 ± 0.1	0.6 ± 0.0	0.2 ± 0.0	4.6 ± 0.4	0.8 ± 0.1	0.3 ± 0.0	0.9 ± 0.1
	10 postmenopausal women, age 51-70	14.5 ± 0.5	3.0 ± 0.2	1.1 ± 0.1	0.2 ± 0.0	11.6 ± 0.6	1.2 ± 0.0	0.8 ± 0.0	1.8 ± 0.1
Pediatric population									
Adriaansen et al. (2024) (44)	62 boys and girls age 0-5	0.57 [<0.35-4.79]	0.21 [<0.05-1.15]	0.04 [<0.03-0.18]	<0.04 [<0.04-0.05]	0.61 [0.18-2.04]	0.10 [<0.09-0.31]	<0.04 [<0.04-0.06]	0.10 [<0.04-0.66]
	133 boys age 0-10 and girls age 0-9	1.89 [<0.35-14.1]	0.48 [0.05-1.64]	0.09 [<0.03-0.30]	<0.04 [<0.04-0.11]	1.00 [0.21-3.72]	0.15 [<0.09-0.49]	0.04 [<0.04-0.15]	0.27 [<0.05-0.99]
	52 boys age 11-17	10.5 [2.54-27.7]	1.59 [0.41-3.32]	12.1 [0.10-21.5]	0.78 [0.05-1.59]	2.27 [0.62-2.27]	0.40 [0.10-0.91]	0.16 [0.05-0.51]	0.99 [0.30-2.35]
	71 girls age 10-17	10.5 [2.54-27.7]	2.13 [0.58-5.39]	0.42 [0.14-0.78]	0.09 [0.04-0.29]	2.27 [0.62-2.27]	0.40 [0.10-0.91]	0.16 [0.05-0.51]	0.99 [0.30-2.35]
Rege et al. (2018) (68)	22 girls age 4-5	1.43 [0.80-1.90]	0.36 [0.29-0.53]	0.13 [0.08-0.17]	n.m.	0.58 [0.37-1.13]	0.27 [0.23-0.37]	0.1 [0.09-0.22]	0.28 [0.24-0.36]
	38 girls age 6-8	2.23 [1.51-4.91]	0.54 [0.36-0.86]	0.17 [0.15-0.19]	n.m.	0.89 [0.66-1.31]	0.38 [0.33-0.48]	0.15 [0.11-0.22]	0.44 [0.34-0.60]
	23 girls age 9-10	4.65 [2.92-6.45]	0.74 [0.66-0.99]	0.22 [0.18-0.28]	n.m.	0.86 [0.58-1.48]	0.43 [0.29-0.56]	0.18 [0.14-0.22]	0.58 [0.47-0.74]

Data are presented as median [interquartile range] or mean ± SEM (as provided by each article). Steroid concentrations are depicted in nmol/L. To convert nmol/L to ng/dL, divide by 0.0349 for DHEA, A4, T, DHT; 0.0331 for 11OHA4, 11KT, 11OHT; 0.0333 for 11KA4. DHEA, dehydroepiandrosterone; A4, androstenedione; T, testosterone; DHT, dihydrotestosterone; 11OHA4, 11-hydroxyandrostenedione; 11KA4, 11-ketoandrostenedione; 11OHT, 11-hydroxytestosterone; 11KT, 11-ketotestosterone.

## 9 Biochemical monitoring using blood samples

Blood withdrawal is a frequently used method for obtaining matrix to evaluate biochemical monitoring as it is feasible across all ages. It provides an immediate snapshot of circulating steroid levels, is widely available, and relatively inexpensive to perform. From a blood sample, either serum or plasma can be obtained. Serum and plasma are not strictly interchangeable for hormone measurements. The use of EDTA to produce plasma improves stability of many labile compounds, such as ACTH and renin (76). Cao et al. (2018) reported significant biases for A4 and 11-deoxycortisol when serum was replaced with EDTA or heparin plasma (77). The authors attributed these effects largely to ion suppression from anticoagulants during MS analysis. Therefore, both serum and plasma can be used for steroid profiling when matrix-specific reference intervals are applied (78–80). However, blood withdrawals are invasive and can induce stress, potentially influencing 17-OHP levels (81). Since venipuncture must be performed in a clinical setting, it imposes logistical burdens for the patient and complicates assessment of diurnal steroid patterns. Obtaining early morning samples, which are critical for evaluating treatment response and adrenal steroid production, can be particularly challenging (28). Therefore, research aims to develop non-invasive alternatives for biochemical monitoring in patients with 21OHD.

## 10 Non-invasive monitoring

Alternative biological matrices have been evaluated for incorporation into 21OHD monitoring practices. These matrices offer opportunities for non-invasive monitoring, each with specific advantages for clinical applications (**Table 2**). Saliva sampling is a highly convenient matrix for routine monitoring, as it enables repeated at-home collection, supporting diurnal steroid profiling. Saliva collection is straightforward and can be collected via passive drool or using commercial devices, making it suitable for most age groups (82). Strong correlations between salivary and serum concentrations have been observed for 17-OHP and A4 (83). A notable distinction is that saliva reflects only the biologically active, free fraction of steroids, whereas serum assays mostly quantify the sum of both free and protein-bound forms (84). Protein-bound steroids are unable to traverse salivary gland membranes, while the small, lipophilic free fraction diffuses passively into saliva, resulting in salivary 17-OHP and A4 levels more than ten-fold lower compared with serum levels (84). This characteristic strengthens the utility of saliva for conditions with altered cortisol-binding protein levels, such as pregnancy, oral contraceptive use, liver disease, or critical illness. Interestingly, cortisol is converted to cortisone in the salivary glands due to the high expression level of 11β-hydroxysteroid dehydrogenase (11βHSD) type 2. Both saliva cortisol and cortisone correlate strongly with free- and total cortisol levels (8).

**Table 2:** Comparison of biological matrices for 21OHD monitoring.

	Invasive	At-home sampling	Suitable for neonates	Repeated sampling	Long-term reflection	Diurnal profiling	Cost-effectiveness	Correlation with serum
Serum	+	-	+	-	-	+	+	n.a.
Saliva	–	+	+	+	–	+	+	+ (17-OHP, A4, cortisol, cortisone)
DBS	+/-	+	+	+	-	+	+	+ (17-OHP)
Urine	+/- (burdensome)	+	+/-	+	+/- (24h coverage)	–	- (expensive assays)	+ (pregnanetriol, backdoor metabolites)
Hair	-	+	-	-	+ (weeks-months)	-	+	- (poor direct correlation)

+ indicates a favorable choice to use, - indicates a limitation, +/- denotes moderate or context-dependent performance.

21OHD, 21-hydroxylase deficiency; DBS, dried blood spots; 17-OHP, 17-hydroxyprogesterone; A4, androstenedione.

Urine profiling using LC-MS/MS or gas-chromatography mass-spectrometry (GC-MS) can be valuable for diagnostics and enables assessment of treatment compliance, since excess or insufficient GC dosing is reflected in urinary metabolite levels (85). Analysis of a urinary steroid profile collected over a longer period of time, for example 24-hours, provides a cumulative representation of steroid metabolism. Since metabolite excretion over the day is pooled into one sample, diurnal patterns cannot be detected. Morning spot urine has been explored as an approach for periodical monitoring, though it does not accurately capture the early morning steroidogenic state, given the time lag introduced by systemic metabolism and excretion (28, 85). Pregnanetriol, a metabolite of 17-OHP, serves as a useful biomarker for treatment monitoring, as its concentrations are elevated in poorly controlled patients with 21OHD (86). Pregnanetriol:tetrahydrocortisol ratios could improve diagnostic discrimination between CAH subtypes (87). Thus, urine steroidomics hold particular value for diagnostics, while other matrices, for instance saliva, are more preferred for routine disease and treatment monitoring.

Steroid analysis in scalp hair offers an even longer-term insight into GC and androgen exposure over periods of weeks to months. Given that scalp hair grows approximately 1 cm per month, consecutive hair segments can be analyzed to reconstruct steroid exposure over a longer time span. Waaijers et al. (2023) detected elevated levels of A4, 17-OHP, and T in hair of CAH patients, with 17-OHP levels differentiating between different CAH subtypes (88). Hair was analyzed in 3 cm segments with averaged steroid levels, which reduces errors from misalignment but limits detection of short-term hormonal fluctuations (89). Heterogeneity in hair structure, growth rate, and cosmetic treatments may introduce matrix-specific effects, thereby influencing steroid measurements. Moreover, scalp hair is a prerequisite for analysis, making this method unsuitable for NBS. Nonetheless, hair analysis could be a useful measure for long-term monitoring in settings with limited access to clinical laboratories.

Dried blood spots (DBS) may provide a practical, minimally-invasive technique for diagnostic screening, especially in remote or resource-limited areas (90). DBS sampling is inexpensive, can be performed at home, requires a small volume of blood, and permits multiple collections throughout the day to generate daily profiles (40). Though, large intra-assay CV at low steroid levels limits the use of DBS for routine monitoring, making DBS more appropriate for diagnostic purposes, where steroid concentrations are generally higher (91).

## 11 Effect of timing of sampling

Timing of sample collection is critical, as adrenal steroid concentrations are influenced by circadian rhythms and dose administration schedules. In well-controlled patients, adrenal steroidogenesis follows a diurnal pattern driven by ACTH secretion, with peak levels of 17-OHP and A4 in the early morning and nadirs in the evening (24). Therefore, early morning steroid levels represent the maximal circulating concentrations, and the timing of sample collection must be carefully aligned with this period to accurately capture peak levels. To assess whether the circadian rhythm is properly established, a daily steroid profile in the morning, afternoon, and evening provides the most informative data, although the ability to obtain such a profile depends on the chosen sample matrix.

In female patients with a menstrual cycle, particular attention should be drawn to steroid production during the cycle, as hormone levels fluctuate during different phases. In the follicular phase, progesterone levels are low and estrogen predominates, with 17-OHP and A4 largely reflecting adrenal production. During the luteal phase, progesterone and 17-OHP concentrations rise, while A4 peaks mid-cycle, indicating gonadal contribution to the measured 17-OHP and A4 levels (92, 93). These cyclical variations show the importance of consistent timing of sample collection within the menstrual cycle. Consequently, early follicular phase sampling is often recommended to minimize gonadal influence and ensure accurate monitoring of adrenal steroidogenesis (4, 36).

## 12 Effect of glucocorticoid replacement therapy on biochemical monitoring

HC is currently the most commonly prescribed therapy for children with 21OHD due to its relatively limited suppressive effect on linear growth. Due to its short half-life, partial destruction by gastric acidity, and first-pass metabolism, multiple daily doses are required to maintain adequate cortisol levels and effectively suppress excess adrenal androgens (10, 94). This pharmacokinetic profile requires careful timing of biochemical monitoring, as 17-OHP and A4 levels fluctuate significantly throughout the day in relation to dosing of HC. As a result, serial collection of samples is required to optimally assess disease control. Morning administration of HC causes a rapid decline in adrenal androgens: 17-OHP decreases by approximately 90% within 3 hours, and A4 by 70% within 4 hours (95). A study of 57 children with 21OHD found that monitoring serum biomarkers before medication intake, which better reflects overnight cortisol and androgen exposure, was associated with improved final height, likely by enabling more precise therapy adjustment (96). This shows that early morning samples should be collected prior to medication intake. Although some clinicians continue to favor reverse circadian therapy, where the highest GC dose is given in the evening, this approach has been associated with metabolic complications, including insulin resistance (97). Although no significant differences in average daily 17-OHP have been observed between circadian and reverse circadian regimens, these regimens produce distinct exposure patterns of both 17-OHP and A4 throughout the day (98–100). Accordingly, international consensus guidelines recommend that biochemical biomarkers should be measured at consistent times relative to both the medication schedule and time of day (11).

Long-acting GCs (prednisone, dexamethasone) show slower clearance and prolonged activity, allowing for fewer daily administrations. While this simplifies dosing schedules, these agents are generally not recommended during childhood due to their potent GC effects and increased risk of adverse effects, especially growth suppression (11). Prednisone shows minimum decreased 17-OHP and A4 levels approximately 4-5 hours after administration, while maxima appear between 9-10 hours. With dexamethasone, minima and maxima are shifted later, occurring around 9 and 16 hours post-dose, respectively (101). These kinetics show the importance of precise timing of sampling in relation to GC type and intake. Modified-release HC (MRHC, Efmody^®^) was developed to better mimic physiological cortisol rhythm and is approved for adolescents aged ≥ 12 years, though pediatric data remain limited. The regimen typically consists of two-thirds of the dose at bedtime to reproduce the nocturnal cortisol rise and early-morning peak, followed by one-third in the morning to sustain daytime cortisol availability. Compared with conventional HC, MRHC produces flatter 24-hour androgen profiles and improved overall androgen control. Consequently, a single morning measurement of 17-OHP or A4 is generally sufficient for monitoring, reducing the need for multiple daily samples (4, 102, 103). In summary, GC selection directly influences the timing of sample collection for accurate biochemical monitoring.

## 13 Future biomarkers

### 13.1 11-oxygenated androgens

Over the past decade, 11OA have gained increasing attention as clinically relevant biomarkers in patients with 21OHD. These steroids include 11β-hydroxyandrostenedione (11OHA4), 11-ketoandrostenedione (11KA4), 11β-hydroxytestosterone (11OHT), and 11-ketotestosterone (11KT). Among these, 11OHA4 and 11OHT are considered adrenal-specific, originating from CYP11B1-mediated hydroxylation of A4 and T, as higher concentrations are found in adrenal compared to ovarian veins, and considering their responsiveness to cosyntropin stimulation (104). Furthermore, 11OA are undetectable in patients with CYP11B1 deficiency, adrenal insufficiency, or after adrenalectomy, while levels remain stable after oophorectomy (105). 11OHA4 represents the principal 11OA precursor, which can be converted to 11KA4 by renal 11βHSD2, and subsequently to 11KT by aldo-keto reductase family 1 member C3 (AKR1C3) in peripheral tissues (106, 107). AKR1C3 also catalyzes the conversion of A4 to T, moreover it has greater catalytic efficiency for 11OA activation (36). 11KT activates the androgen receptor with a potency comparable to T, and can be further metabolized to 11KDHT, a DHT analogue (68).

During gestation, enzymes required for 11OA synthesis are already expressed in fetal tissues and the placenta (37). Although the precise role of 11OA in human development remains to be clarified, their presence suggests a previously unrecognized source of intrauterine androgen exposure. Interestingly, early neonatal steroid profiles are dominated by 11OHA4 and 11-ketoprogesterone, often exceeding concentrations of A4 and T (37). Several 11-oxygenated steroids, such as 11KT, 21-dF and 21-deoxycortisone show particular diagnostic specificity for 21OHD, and improve distinction between false-positive and false-negative NBS results when used as second-tier markers (108–110). The clinical relevance of 11KT is highlighted by its pivotal role in both normal and premature adrenarche, as well as by the fact that it represents the dominant androgen in postmenopausal women (44, 68, 69, 111). This has prompted investigation into whether 11OA are major contributors to 21OHD disease manifestation. Multiple studies have shown significantly elevated 11OA concentrations in patients with 21OHD compared with healthy controls (112). Moreover, a weak correlation between classic androgens and their 11-oxygenated metabolites indicates that solely measuring conventional markers underestimates the total androgen pool (44).

11OA display a circadian rhythm similar to 17-OHP and A4, but show smaller fluctuations directly after medication administration, increasing their robustness for monitoring (72). In females, the diurnal pattern of 11OA persists across the menstrual cycle. Therefore, for measuring 11OA, the time of sampling during the menstrual cycle does not have to be taken into account (36, 107). Analytical attention should be drawn to enzymatic and non-enzymatic conversions, as cortisol and cortisone may convert to 11OHA4 and 11KA4, a process that is highly temperature-sensitive, and already observed at room temperature but was negligible at -20 °C (69, 113). Therefore, a controlled temperature during sample processing is important for acquiring accurate results. Unlike classic biomarkers, circulating 11OA remain relatively stable with age owing to preserved CYP11B1 expression in the zona fasciculata despite involution of the ZR (69, 114). In some patients whose classical androgens were within normal range, elevated 11OHT and 11KT levels have been observed. Jha et al. showed 2.5-fold higher 11KT concentrations in patients with substantially increased 17-OHP but normal A4 levels, with more than a quarter of these cases being clinically poorly controlled (34).

Thus, 11OA quantification may offer significant advantages for both diagnosis and long-term follow-up in patients with 21OHD. Their measurement may discriminate between adequate and inadequate disease control, particularly in cases where traditional biomarkers provide discordant results.

### 13.2 Markers to evaluate metabolic risk

Another example where future biochemical biomarkers may complement clinical parameters is in assessing the effects of high body weight. Increased adipose tissue and insulin resistance upregulate AKR1C3 activity, enhancing peripheral 11KT production (36). In girls experiencing premature adrenarche, those with BMI above the 85^th^ percentile exhibit higher 11KT levels than normal-weight peers (68). In addition to 11OA, altered leptin signaling further exacerbates obesity in 21OHD. Leptin, a hormone predominantly produced by adipocytes in white adipose tissue, regulates food intake and body mass. Both GC exposure and obesity induce leptin resistance, typically characterized by hyperleptinemia and reduced soluble leptin receptor (sOB-R) levels (115). In 21OHD, sOB-R levels are decreased independently of BMI, suggesting disease-specific suppression of sOB-R that may accelerate leptin clearance, hence resulting in satiety dysregulation and promoting weight gain. Hyperandrogenism reinforces this process, since androgens could suppress sOB-R expression (116). Moreover, leptin and insulin can directly stimulate adrenal CYP17 17,20-lyase and 3β-HSD activity, further enhancing androgen production (117). Hence, maintaining a low BMI is crucial to limit androgen excess, with incorporation of leptin, sOB-R, and 11OA measurements to optimize the evaluation of metabolic risk. However, in low-resource settings, limited access to biomarker testing forces clinicians to rely solely on clinical parameters to guide therapy.

### 13.3 Markers to estimate glucocorticoid receptor sensitivity

While current biomarkers provide highly effective strategies for biochemical monitoring of treatment response in patients with 21OHD, achieving optimal GC dosing may still remain challenging in a particular subset of patients. Besides inter-individual variability in GC pharmacokinetics, differences in the response to GC therapy can also be an important contributor. Emerging evidence suggests this may be partly driven by differences in GR sensitivity, explaining why certain patients require higher GC dosages than others (118). Therefore, assessment of GR sensitivity, alongside careful interpretation of biochemical markers, could enable more personalized starting dose adjustments and help to prevent both over- and undertreatment.

Several factors influence GR sensitivity, including genetic polymorphisms. The GR is a nuclear receptor encoded by the *NR3C1* gene on chromosome 5, and serves as a central mediator of GC effects on gene expression, metabolism, and stress responses. Genetic variants in *NR3C1* modulate GR function and clinical response to GCs in 21OHD (119). For example, the N363S polymorphism has been associated with milder virilization in females with 21OHD, while the A3669G variant increases GR-β expression, a dominant-negative inhibitor of GR, and has been linked to adverse metabolic profiles in pediatric 21OHD patients (120, 121). Additionally, a trend towards higher A4 levels was observed in carriers of the common BclI variant (122). These findings collectively suggest that GR genotype may be a relevant determinant of both hormonal control and metabolic risk in patients with 21OHD.

The altered hormone balance in 21OHD may also indirectly affect GC activity by influencing GC availability. Elevated concentrations of precursor steroids may compete for corticosteroid-binding globulin, possibly increasing free cortisol (123). Despite lower total cortisol in untreated patients with 21OHD, free cortisol levels are similar to controls, which is relevant as only free cortisol activates GR-mediated signaling (124, 125). Additionally, enzymes such as 11βHSD1/2 regulate tissue-specific GC activation in organs like liver, adipose tissue, and kidney. 11βHSD1 is regulated by androgens and estrogens, possibly contributing to sex-specific differences in GC responses (126). P-glycoprotein, encoded by *ABCB1*, also affects GC availability by reducing intracellular concentrations. A pilot study in children with classic CAH identified several pharmacogenetic links between *ABCB1* variants, encoding for efflux transporters that reduce intracellular GC concentrations (127). These results highlight the complex regulatory network linking GR activity, local GC availability and systemic outcomes.

In addition to genetic and enzymatic factors, biomarkers of transcriptional GR activity have been proposed as tools for monitoring GC responsiveness. For instance, circulating FKBP5 mRNA has emerged as a potential biomarker in patients with Addison’s disease, reflecting GR-mediated transcriptional activity (128). Similar transcriptional readouts could potentially be applied in 21OHD to provide real-time, functional insights into receptor sensitivity. Taken together, assessing GR sensitivity through genetic profiling, functional assays, or transcriptional biomarkers such as FKBP5 could serve as a valuable future strategy to personalize GC therapy in patients with 21OHD.

## 14 Discussion

A variety of monitoring practices are used to evaluate treatment response in patients with 21OHD, i.e., analytical methods and instrumentation, pre-analytical handling, matrix effects, and applied reference ranges, leading to differences in the interpretation of biochemical markers. Awareness of these sources of variation is therefore essential for advancing toward optimal monitoring of patients with 21OHD.

The most effective approach to assess treatment response integrates monitoring of clinical parameters with biochemical measurements, with clinical context serving as the primary guide and biochemical data used to corroborate clinical observations. Clinical evaluation consists among others of growth, bone age, blood pressure and pubertal development. Biochemical monitoring can be performed with a set of biomarkers. Solely measuring 17-OHP is not advised, as there is not always an optimal concordance with the clinical phenotype (129). Multiplexing of steroids provides a more comprehensive steroid profile (130). Often, A4 combined with 17-OHP could yield a more informative view on hyperandrogenism and replacement effects. However, a subset of patients demonstrates discrepancies between 17-OHP and A4 levels (34). Biochemical monitoring of 17-OHP and A4 is further complicated by the fact that both biomarkers are not adrenal-specific. Within this context, 11OA have emerged as promising biomarkers in 21OHD management. Their adrenal origin and potent androgenic activity, in particular 11KT, highlight their potential value in 21OHD monitoring (69, 104). Apart from 21OHD, involvement of 11OA is observed in other endocrine and metabolic disorders. Elevated concentrations have been reported in PCOS, where levels were nearly five-fold higher than in healthy controls, as well as in castration-resistant prostate cancer, and Cushing syndrome (44, 131–134). Also, 11OA might be helpful to distinguish between adrenal and ovarian contributions to hyperandrogenism and fertility disturbances. However, the integration of 11OA in routine clinical care is hampered since standardized assays and protocols are currently lacking.

An important consideration of biochemical monitoring is matrix selection. Serum is often used for diagnostic confirmation and acute assessment, but alternative matrices offer distinct advantages for non-invasive monitoring. Saliva facilitates repeated sampling and is particularly suited for home-based monitoring (35, 83). Recent progress in defining salivary reference intervals represents an important step forward for non-invasive monitoring (44, 83). DBS or hair do not capture the same informative profile as saliva, though these matrices might be useful in remote or resource-limited setting (40, 88, 90). Therefore, each matrix expands opportunities for individualized monitoring when applied in the appropriate clinical context. Thus, matrix choice depends on clinical question, patient age, available resources, and biomarker-of-interest.

A further limitation of current practice is that most published reference intervals are derived from adult populations, while pediatric data remain sparse. This is problematic given that steroid physiology undergoes profound changes during fetal and neonatal life, adrenarche, and puberty (24, 37, 135). Hence, longitudinal, multicenter studies are essential to define robust age-specific reference ranges for both classic and novel biomarkers. The type of GC therapy also influences interpretation. While prednisolone and dexamethasone more effectively suppress androgens than HC, their use in childhood is limited by adverse effects on growth (29).

In addition to analytical and center-specific differences, patient-specific factors may also contribute to variation in biomarker levels. For instance, type of mutation might affect treatment response through altered enzyme activity and consequent shifts in steroid profiles (4). Similarly, individual variation in GR sensitivity could influence treatment efficacy, suggesting that examining receptor sensitivity reduces instances of overtreatment and allows for more tailored dosing. This could explain why certain 21OHD patients require different GC doses to achieve equivalent levels of disease control. Demographic differences, country-specific prevalence patterns, and differences in treatment compliance also add to complexity. Nevertheless, current evidence indicates that center-specific components remain the dominant source of variability.

In conclusion, variation in monitoring practices contributes to differences in measured biomarker levels across centers. These differences emphasize the importance for interpreting the results in the context of patient age, type of medication, sample matrix and the analytical method. Method-specific reference intervals should be traceable to higher-order standards, to ensure that reference values and clinical decision limits are appropriate for their particular measurement setup. Participation in External Quality Assessment Schemes allows laboratories to evaluate the performance of their methods relative to other centers (136). Clinicians must be aware of the inherent limitations of the assays and incorporate this knowledge into the overall interpretation of clinical and biochemical data. It is therefore crucial that clinicians are familiar with the methodology used for steroid measurements in their own center. A thorough understanding of variations in monitoring practices and the application of assay-, age-, and matrix-specific reference intervals are essential to advancing harmonized, optimized, and individualized management of 21OHD.

## Glossary

11βHSD: 11β-hydroxysteroid dehydrogenase
11KA4: 11-ketoandrostenedione
11KDHT: 11-ketodihydrotestosterone
11KT: 11-ketotestosterone
11OHA4: 11β-hydroxyandrostenedione
11OHT: 11β-hydroxytestosterone
11OA: 11-Oxygenated Androgens
17-OHP: 17-hydroxyprogesterone
21-dF: 21-deoxycortisol
21OHD: 21-Hydroxylase Deficiency
A4: Androstenedione
ACTH: Adrenocorticotropic Hormone
AKR1C3: Aldo-Keto Reductase Family 1 Member C3
BMD: Bone Mineral Density
CAH: Congenital Adrenal Hyperplasia
CV: Coefficient of variation
DBS: Dried Blood Spot
DHEA: Dehydroepiandrosterone
DHEAS: Dehydroepiandrosterone Sulfate
DZ: Definitive Zone
FC: Fludrocortisone
FSH: Follicle-Stimulating Hormone
FZ: Fetal Zone
GC: Glucocorticoid
GC-MS: Gas Chromatography–Mass Spectrometry
GnRH: Gonadotropin-Releasing Hormone
GR: Glucocorticoid Receptor
HC: Hydrocortisone
HPG: Hypothalamic–Pituitary–Gonadal axis
LC-MS/MS: Liquid Chromatography–Tandem Mass Spectrometry
LH: Luteinizing Hormone
MC: Mineralocorticoid
NBS: Newborn Screening
PRA: Plasma Renin Activity
PRC: Plasma Renin Concentration
SV: Simple Virilizing
SW: Salt-Wasting
T: Testosterone
TART: Testicular Adrenal Rest Tumor
TZ: Transition Zone
ZR: Zona Reticularis.
